# Evaluating an Eight-Week Therapeutic Swimming Program in Children with Autism Spectrum Disorder: A Mixed-Methods Study from Romania

**DOI:** 10.3390/children12121646

**Published:** 2025-12-03

**Authors:** Stefan Alecu, Gheorghe Adrian Onea

**Affiliations:** Department of Physical Education and Special Motricity, Faculty of Physical Education and Mountain Sports, Transilvania University, 500068 Brasov, Romania

**Keywords:** autism spectrum disorder, adapted physical activity, therapeutic swimming, aquatic therapy, Romanian, parental perception, child development

## Abstract

**Highlights:**

**What are the main findings?**

- Children with autism showed significant improvements in well-being, physical activity, attention, learning, and self-confidence, with the greatest gains occurring after the 8-week adapted therapeutic swimming program.
- Parent-reported outcomes confirmed not only measurable progress but also meaningful daily-life benefits, including calmer behavior, improved sleep, and increased social interaction.

**What are the implication of the main findings?**

- Adapted therapeutic swimming can be integrated as a feasible and effective component of multidisciplinary rehabilitation for children with autism, complementing behavioral therapies such as ABA.
- Expanding access to aquatic programs in Romania may address the current lack of adapted physical education options, improving both child outcomes and family well-being.

**Abstract:**

**Background/Objectives:** In Romania, therapeutic program programs for children with autism spectrum disorder (ASD) focus mainly on behavioral and educational approaches, with limited integration of adapted physical activity (APA). Therapeutic swimming may provide complementary benefits, addressing both physical and psychosocial challenges. This study explored parent-perceived changes across an adapted therapeutic swimming program, following ABA therapy, on the well-being, learning, attention, physical activity, and social functioning of children with ASD. A custom-developed parent-report tool (PPQ-Autism-Swim) was used to measure perceived changes across key developmental domains. **Methods:** Thirty-nine children with ASD were recruited, of whom 36 completed the full 8-week swimming intervention. Parent-report questionnaires, developed by behavioral therapists under the supervision of a clinical psychologist, were administered at three time points: 8 weeks before swimming (T − 8), immediately before swimming after Applied Behaviour Analysis (ABA) therapy (T0), and 8 weeks after swimming (T + 8). The questionnaire assessed five subscales: general well-being, physical activity and energy, attention and focus, learning and cognitive progress, and self-confidence and social behaviors. Data were analyzed using descriptive statistics, repeated-measures ANOVA, and thematic analysis of qualitative parental feedback. ABA therapy served as a behavioral baseline, allowing comparison with subsequent gains from swimming. **Results:** Parents reported higher scores across time points, suggesting perceived changes in several domains. From T − 8 to T0, moderate gains followed ABA therapy, while from T0 to T + 8, therapeutic swimming was associated with improvements. At T + 8, 35 of 36 children showed measurable progress, particularly in physical activity regulation and self-confidence. Parental feedback emphasized calmer behavior, improved sleep, increased social interaction, and greater pride in new skills. Reliability testing indicated good to excellent internal consistency (Cronbach’s α = 0.78–0.91). **Conclusions:** Therapeutic swimming appears to be an effective and motivating form of adapted physical education, complementing behavioral therapies and addressing multiple developmental challenges in children with ASD.

## 1. Introduction

Autism Spectrum Disorder (ASD) is a neurodevelopmental condition characterized by persistent difficulties in social communication and restricted, repetitive patterns of behavior or interests. Global prevalence estimates indicate that approximately one in every hundred children is affected, although recent increases in diagnostic rates reflect improved awareness and screening practices [1]. Children with ASD frequently experience challenges related to communication, behavioral regulation, sensory hypersensitivity and high levels of caregiver stress [2]. Standard therapeutic approaches, including Applied Behavior Analysis (ABA), speech therapy and occupational therapy, aim to improve communication, self-regulation and daily functioning. However, research increasingly shows that physical activity provides complementary developmental benefits for children with ASD. Regular exercise improves gross motor coordination and reduces hyperactivity [3,4].

It supports emotional regulation, strengthens attention and facilitates social engagement through turn-taking, joint attention and cooperative tasks [5,6]. Physical activity also enhances cardiovascular fitness, muscle strength and energy regulation. Studies by Pan et al. (2017) and Sowa and Meulenbroek (2012) demonstrate improvements in executive functions such as cognitive flexibility, working memory and inhibitory control following structured exercise programs [4,7]. These benefits are especially relevant for children with ASD, who often present co-occurring challenges across physical, cognitive and socio-emotional domains.

Adapted physical activity (APA) provides structured movement opportunities tailored to the abilities and needs of children with developmental disorders. APA programs aim to improve motor skills, sensory integration and self-regulation, functioning as an important complement to behavioral and educational therapies. Structured movement activities help integrate sensory input, supporting attention and reducing anxiety by promoting self-regulation of arousal levels [3,5]. Research shows that structured physical activity programs improve executive functions in children with ASD, including planning, working memory, and response inhibition.

Among the few adapted physical modalities accessible, aquatic interventions hold promise. The water environment provides unique sensory and motor experiences, reduces gravitational constraints, and offers both relaxation and stimulation, making it especially suitable for children with ASD who often face challenges with coordination, regulation, and participation in traditional sports. In this context, it is important to distinguish between two approaches commonly used: aquatic therapy and therapeutic swimming [8,9,10,11,12,13]. According to definitions from the American Physical Therapy Association (APTA, 2022), aquatic therapy targets specific clinical outcomes like sensory regulation, mobility, and motor control through structured, hands-on techniques. The therapeutic swimming refers to structured instruction in swimming skills provided in a supportive but non-clinical setting. It assumes that the child can understand and respond to verbal prompts and participate more independently. The focus is on promoting physical activity, attention, social engagement, and confidence through consistent routines and skill progression. This distinction is important for selecting appropriate methods based on the child’s developmental readiness, behavioral profile, and level of autonomy providing a flexible framework for adapting aquatic interventions to the child’s developmental stage and needs [14,15].

Aquatic therapy refers to therapist-directed sessions for children who are not yet able to follow verbal instructions. The therapist maintains physical control and uses hands-on techniques to guide movement and ensure safety. This approach is often used in early intervention or with children with significant developmental delays [8,10]. In contrast, therapeutic swimming is used when the child can understand verbal cues and actively participate in learning swimming skills. It emphasizes independent movement, guided prompting, and skill acquisition in a structured environment [11,12].

Importantly, the flexibility of alternating between aqua therapy (therapist-led) and therapeutic swimming (child-engaged with prompting) allows interventions to be tailored to each child’s abilities and developmental level. In contrast with sports therapy, aquatic therapy introduces additional therapeutic elements that land-based sports cannot provide. The buoyancy of water reduces joint pressure and body weight, making movement easier and less fatiguing. Hydrostatic pressure offers constant, calming sensory input that can help children with ASD manage anxiety and sensory overload. The warmth of the water promotes muscle relaxation and reduces physical tension, which supports better motor coordination and body awareness [10,11]. Caputo et al. (2018), Zanobini and Solari (2019), and Alaniz et al. (2017) [10,11,12] found that aquatic therapy improved social communication, coordination, and attention in children with autism. More recently, Marzouki et al. (2022) [15] reported significant gains in emotional regulation and physical control after aquatic interventions. Together, these features make aquatic therapy especially suitable for children with ASD, who often face challenges with movement planning, postural control, and adapting to sensory input. These sensory-motor benefits are not present in traditional sports environments, which may be overstimulating or physically demanding.

Although aquatic therapies on autistic children have been studied in countries such as the United States, Canada, Australia, and Western Europe, research from Eastern European contexts, and Romania in particular, remains limited. International studies consistently show that swimming and aquatic therapy improve motor coordination, cardiovascular fitness, self-regulation, and social behaviors in children with ASD [2,16,17].

Another major challenge in the Romanian context is the high prevalence of comorbidities among children with ASD and other special needs [18,19,20]. Pasco et al. (2014) also highlighted that Romanian services for children with ASD remain fragmented, often depending on NGOs rather than coordinated public systems [21].

Against this background, therapeutic swimming emerges as a promising and underutilized solution. It provides not only physical exercise but also a structured educational context where children can practice attention, self-control, and social interaction in an enjoyable environment [22]. Unlike many therapies that children may experience as demanding or stressful, swimming is often perceived as fun, increasing motivation and participation. This adaptability makes swimming a uniquely inclusive form of adapted physical education and therapy, with the potential to address the multifaceted challenges of thetherapeutic program in Romanian children with autism and other special needs [23,24].

To provide a structured foundation for the study, we drew upon Bronfenbrenner’s Ecological Systems Theory (1979) and Newell’s Constraints Model of Motor Development (1986) as guiding frameworks [25,26]. These models emphasize the dynamic interaction between the individual, the environment, and the task. In the context of autism therapy, this suggests that structured aquatic environments can serve as a powerful setting where individualized physical tasks (swimming exercises) interact with the child’s unique developmental profile and the therapeutic support provided.

Additionally, Sensory Integration Theory (Ayres, 1972) informs our understanding of how water-based sensory experiences such as hydrostatic pressure, buoyancy, and temperature may help regulate arousal levels and support attentional control, particularly in children with ASD who often face sensory modulation difficulties [27]. These frameworks collectively support the rationale that swimming is not only a form of physical exercise but also a multisystem intervention capable of enhancing self-regulation, executive functioning, emotional balance, and social interaction.

The present study builds on these theoretical underpinnings to explore how adapted therapeutic swimming can reinforce behavioral gains from ABA and address developmental challenges holistically.

Infrastructure is also a major limitation. Few swimming pools are accessible for children with disabilities, and most do not have staff trained in adapted aquatic instruction or behavioral management. Many families must travel long distances or cover program costs themselves, creating strong social inequities. These conditions make the implementation of structured aquatic interventions both innovative and logistically demanding in the Romanian context.

The aim of this study was to describe parent-perceived changes across an eight-week therapeutic swimming program, situated within the children’s broader therapeutic trajectory, following a structured period of Applied Behavior Analysis (ABA), on multiple domains of functioning in children with autism spectrum disorder (ASD). Specifically, we investigated changes in well-being, attention, learning, physical activity, and social behaviors as reported by parents. We started from the assumption that the participation in a structured one-month therapeutic swimming program will lead to measurable improvements in autistic children aged 8–16 years, as reported by their parents, specifically reflected in enhanced quality of life, increased self-confidence, improved attention and learning progress, greater engagement in daily activities, and reduced hyperkinetic behaviors compared to pre-intervention levels.

### Terminology and Conceptual Definitions

In this study, several terms are used to describe physical and aquatic-based interventions. These distinctions help clarify the scope and goals of the swimming program described in this study.

- Aquatic therapy refers to therapist-led water-based sessions in which the child receives full physical guidance and support, typically used when verbal instruction is not yet effective.
- Therapeutic swimming describes structured sessions where the child can follow verbal cues and actively practice swimming skills with minimal assistance.
- Aquatic therapies is used as an umbrella term that includes both approaches.
- Sports therapy refers to land-based physical activity programs designed for rehabilitation or skill development.
- Adapted physical education covers any structured physical activity program tailored to individuals with disabilities, including both land and water-based interventions.

## 2. Materials and Methods

### 2.1. Participants

The study was conducted in collaboration with two non-governmental organizations (NGOs) in Brașov, Romania, which provide therapy for individuals with special needs. A total of 76 children with ASD were enrolled in the NGOs. Consent was obtained from the legal guardians of 58 children. Of these, 39 children met inclusion criteria and participated in the intervention, (28 males, 71.8%; 11 females, 28.2%), aged 8–16 years (mean age = 11.3 ± 2.4 years). Most children (79.5%) were enrolled in mainstream schools, while 20.5% attended special schools. None of the children had participated previously in structured swimming or aquatic therapy programs. However, six children had limited recreational exposure to swimming, which was not considered formal training. Comorbidities: Intellectual disability: 71.8%, language disorder: 56.4%, developmental delay: 46.1%, hormonal imbalances: 35.9%, epilepsy: 5.1%. All children had a confirmed diagnosis of ASD established by a child psychiatrist using DSM-5 criteria and ADOS-2 assessments. All participating children had been engaged in Applied Behavior Analysis (ABA) as part of their routine therapeutic care prior to the swimming program. ABA was not standardized for this study, and its duration, frequency, and goals varied considerably across families. Reported intensities ranged from approximately 10 to 25 h per week, with therapy durations spanning several months to several years. ABA typically targeted foundational behaviors such as instruction-following, sustained attention, communication skills, and tolerance for structured routines. Because ABA exposure differed across children and was not monitored or modified by the research team, it should be considered a heterogeneous background therapy and a potential confounding factor, rather than a controlled or comparable baseline.

Children were eligible if they had a confirmed ASD diagnosis established by a child psychiatrist using DSM-5 and ADOS-2 criteria, along with at least one comorbid condition (e.g., ADHD, language disorder, developmental delay, or intellectual disability). We acknowledge that requiring completion of ABA therapy may have introduced a selection bias by including children already accustomed to structured routines and verbal instruction. This criterion likely favored participants with higher functional readiness for aquatic engagement, which may limit the generalizability of findings to children with more severe behavioral or cognitive impairments.

Exclusion criteria included severe medical conditions that contraindicated swimming, such as uncontrolled epilepsy or serious cardiac and respiratory disorders, and behavioral difficulties posing safety risks in the pool environment, such as aggression or self-harm. Children were also excluded if their legal guardians did not provide written informed consent.

### 2.2. Study Design and Data Collection Timeline

This study followed a pre-post exploratory design, using repeated measures at three time points to assess within-subject changes over time. We acknowledge the absence of a control group, which limits causal interpretation of the effects observed after the swimming intervention. This longitudinal intervention study was conducted between March 2022 and October 2024. It employed a repeated-measures design with three assessment points. This single-group repeated-measures design allows examination of within-subject score changes across time, but it does not permit causal attribution to the swimming program. Changes may reflect maturation, prior ABA therapy, or parental expectation effects. The questionnaire was developed for this project and has not been formally validated. Results should therefore be interpreted as descriptive and exploratory.

- T − 8 weeks: At the beginning of therapeutic intervention within the NGOs.
- T0: After 8 weeks of psychological and behavioral therapy (Applied Behavior Analysis), immediately prior to initiating therapeutic swimming.
- T + 8 weeks: At the end of the 8-week therapeutic swimming intervention, immediately after the final session. The T0 assessment took place after each child’s routine ABA therapy, but it should not be viewed as an experimental baseline. ABA duration and intensity varied widely, and the research team did not standardize or monitor it. Changes between T − 8 and T0 may reflect ABA-related progress, maturation, or shifts in parent perception. Because ABA was heterogeneous and uncontrolled, it serves as a contextual factor and potential confounder, not a comparison phase. The study therefore cannot isolate the specific contribution of the swimming program. No additional waiting period occurred after the intervention. The final assessment was conducted in the same week as the last swimming session to capture immediate post-intervention outcomes.

**Figure 1:**
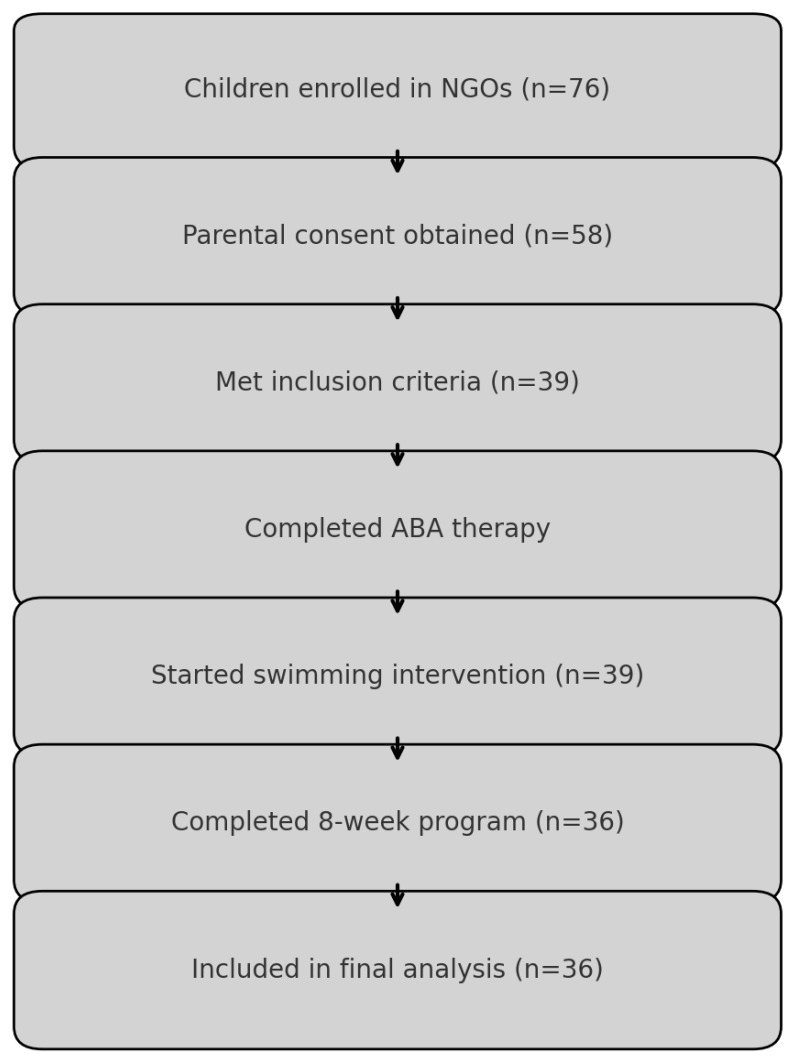
Flowchart of participant recruitment, intervention, and analysis phases.

Figure 1 presents the participant flow from enrollment through ABA therapy, swimming intervention, and final analysis. In addition to quantitative assessment, a qualitative component explored parents’ perspectives on their child’s behavioral, emotional, and social changes. Semi-structured interviews were conducted during the final week of the swimming program (T + 8 weeks) by the child’s behavioral therapist, who was trained in qualitative interviewing and not directly involved in data analysis to minimize bias. Each interview lasted approximately 15–20 min and was conducted face-to-face in a quiet consultation room at the therapy center. Qualitative data were analyzed using an inductive thematic analysis following Braun and Clarke’s six-phase framework. Two researchers independently coded all transcripts line-by-line. Initial codes were generated, compared, and refined iteratively, and discrepancies were resolved through discussion.

The interview guide consisted of six open-ended questions addressing (1) general observations of the child’s behavior after the program, (2) emotional regulation and anxiety, (3) social interaction and communication, (4) motivation and daily routines, (5) physical activity and sleep patterns, and (6) perceived usefulness of the aquatic experience. Parents were encouraged to elaborate freely and provide examples from everyday life. Themes were double coded by two independent researchers. Inter-rater differences were resolved through discussion, ensuring thematic consistency. Coding categories were aligned with the five quantitative domains to facilitate triangulation.

Interviews were audio-recorded with consent, transcribed verbatim, and analyzed using inductive thematic analysis following Braun and Clarke’s six-phase framework [28], which involves (1) familiarization with the data, (2) generating initial codes, (3) searching for themes, (4) reviewing themes, (5) defining and naming themes, and (6) producing the report. Two independent researchers coded the transcripts, compared interpretations, and resolved discrepancies through discussion. Themes were organized to reflect the five functional domains also covered in the quantitative assessment, facilitating triangulation between qualitative and quantitative data.

The design allowed the clinicians to establish a baseline psychological and behavioral profile of each child and to adapt the swimming intervention accordingly. A clinical psychologist supervised the therapeutic plan and provided feedback to the swimming therapist to ensure individualized adaptation.

In this study, Applied Behavior Analysis (ABA) served as an important therapeutic context preceding the aquatic program. All participating children had been enrolled in ABA prior to swimming; however, the duration, intensity, and specific goals of ABA varied across families and were not standardized by the research team. ABA typically focused on developing foundational behaviors such as instruction-following, sustained attention, functional communication, and routine compliance—skills that facilitated participation in structured activities like swimming. Because ABA was ongoing or had recently been completed for many children, it represents a meaningful contextual factor that may influence parent perceptions and the interpretation of changes observed across time. This approach reflects the integration of complementary therapies in multidisciplinary settings and focuses on the potential added value of aquatic intervention in an already structured therapeutic routine.

### 2.3. Instruments and Measures

Parents completed a Custom Parent Perception Questionnaire (PPQ-Autism-Swim) at each assessment point. When both parents were not available, only one primary caregiver (most often the mother) completed the PPQ-Autism-Swim to ensure consistency across participants. Questionnaires were collected from 64 parents representing 36 children. In most families, both parents completed the PPQ-Autism-Swim independently at each time point, and all responses were included in the analysis. This approach allowed us to capture a broader perspective on the child’s behavior and functioning. Consequently, no inter-rater reliability analysis between parents was conducted. This approach was chosen to minimize variability related to different parental perceptions of behavior. The questionnaire was developed by the behavioral therapists of the two organizations, under the coordination of the lead clinical psychologist, who also serves as supervisor for both NGOs., for this study to capture parental perceptions of their child’s well-being and developmental progress. The PPQ-Autism-Swim was developed and administered in Romanian, the native language of all participants and parents. The items were created by Romanian-speaking behavioral therapists, reviewed by a licensed clinical psychologist, and pretested with a small group of parents (n = 10) to ensure clarity and cultural relevance. Item grouping was based on functional domains commonly monitored in ASD interventions (well-being, physical activity, attention, learning, and social behaviors). Items were adapted from constructs represented in widely used parent-report tools such as Vineland-3, PedsQL, SRS-2, and ABC. The aim was to capture everyday behavioral and emotional functioning in a format accessible to caregivers. Based on feedback, minor wording adjustments were made before data collection.

The PPQ-Autism-Swim is an exploratory, non-validated parent-report tool developed specifically for this study. It was used solely to capture perceived changes and is not intended as a psychometrically validated measure of child outcomes. Therefore, the questionnaire was linguistically validated for Romanian use through expert review and pilot testing. It included six sections:

1. General child well-being (happiness, anxiety, curiosity);
2. Physical activity and energy (endurance, hyperactivity, enjoyment of movement);
3. Attention and focus (ability to concentrate, distractibility, following instructions);
4. Learning and cognitive progress (memory, engagement, learning progress);
5. Self-confidence and social behaviors (pride, confidence, interaction, trying new activities);
6. Parent’s perception of impact (optimism, value of routines, perception of swimming benefits).

Theoretical basis and related instruments: The PPQ-Autism-Swim was built to reflect functional domains used in child outcomes research and clinical practice, aligning with adaptive functioning and health-related quality of life frameworks. We mapped items to constructs covered by widely used parent-report tools, including general psychosocial functioning and hyperactivity captured by the Strengths and Difficulties Questionnaire (SDQ), global quality of life domains in the Pediatric Quality of Life Inventory (PedsQL 4.0), adaptive behavior in the Vineland Adaptive Behavior Scales (Vineland-3), social reciprocity in the Social Responsiveness Scale (SRS/SRS-2), and problem behaviors tracked by the Aberrant Behavior Checklist (ABC) (Goodman, 1997; Varni et al., 2001; Sparrow et al., 2016; Constantino & Gruber, 2012; Aman et al., 1985; Kaat et al., 2014) [22,23,24,29,30,31]. Item writing also followed ABA principles on observable behavior and parent-report feasibility, and used motivational structuring consistent with Self-Determination Theory for competence, autonomy, and relatedness during aquatic tasks (Roane et al., 2016; Ryan & Deci, 2000) [32,33] Together, these sources guided item content and scaling and explain the overlap between our subscales and established constructs in child outcomes and ASD assessment. While the PPQ-Autism-Swim was not standardized, its structure drew from validated instruments (e.g., SDQ, Vineland-3, PedsQL), and it demonstrated strong internal consistency (Cronbach’s α = 0.78–0.91) and test–retest reliability (ICC = 0.82–0.89). These indicators support its use in capturing intra-individual changes. Responses were rated on a 5-point Likert scale (1 = not at all true, 5 = completely true). One item (C2: “My child is easily distracted”) was reverse scored.

The sixth section, Parent’s perception of impact, captured the parents’ subjective evaluation of the program’s benefits and their optimism regarding its influence on daily routines. Because these items reflected parental attitudes rather than direct child outcomes, this section was analyzed qualitatively and incorporated into the thematic synthesis (Section 3.1) rather than the quantitative results.

The Parent Perception Questionnaire (PPQ-Autism-Swim) was designed specifically for this study to capture parent-reported changes in their child’s emotional, behavioral, and functional development across intervention phases. The tool was created in Romanian by a team of behavioral therapists under the supervision of a licensed clinical psychologist. It was pretested with 10 parents to ensure clarity and cultural relevance. Based on feedback, minor adjustments were made before full deployment.

The questionnaire contains six subscales:

1. General well-being;
2. Physical activity and energy;
3. Attention and focus;
4. Learning and cognitive progress;
5. Self-confidence and social behaviors;
6. Parent’s perceived impact.

Each item was rated on a 5-point Likert scale (1 = not at all true, 5 = completely true). One item (C2: “My child is easily distracted”) was reverse scored. Subscale scores were calculated as means, with higher values indicating more positive outcomes.

Psychometric testing showed good to excellent internal consistency across all subscales, with Cronbach’s α values ranging from 0.78 to 0.91. Test–retest reliability was assessed in a subset of ten parents and yielded intraclass correlation coefficients (ICC) between 0.82 and 0.89. Although the PPQ-Autism-Swim has not yet undergone formal validation against standardized instruments, its structure was aligned with functional domains from the SDQ, PedsQL 4.0, Vineland-3, SRS-2, and ABC. Its development was also informed by principles of applied behavior analysis and Self-Determination Theory to ensure ecological and motivational relevance.

To assess perceived changes in the children’s quality of life and behavior, questionnaires were administered at three time points (eight weeks before the start of the program, immediately before the intervention, and eight weeks after completion). Importantly, the questionnaires were completed independently by both parents of each child, providing a more balanced and reliable parental perspective. Prior to data collection, parents were asked to confirm that they spent sufficient daily time with their child to accurately evaluate their behaviors and progress. All participating parents declared that they were involved in their child’s daily care and activities for more than eight hours per day, ensuring that their responses reflected consistent, firsthand observations of the child’s psychological state, learning, daily functioning, and social behaviors.

Although, to assess the impact of the intervention, a structured parent-report questionnaire detailed in Appendix A was developed by a team of behavioral therapists and supervised by a clinical psychologist. This tool measured changes in five areas relevant to autism intervention: general well-being, physical activity and energy, attention and focus, learning and cognitive progress, and self-confidence and social behavior. The inclusion criteria focused on children with confirmed ASD diagnoses who had already completed a structured ABA program, ensuring a consistent baseline of behavioral functioning. In addition to quantitative assessments, qualitative feedback was collected from parents at the end of the swimming program (T + 8 weeks). Each child’s behavioral therapist conducted a semi-structured interview lasting approximately 10–15 min. Parents were invited to describe perceived changes in their child’s emotional regulation, social behavior, daily functioning, and physical well-being. Interviewers used a predefined set of guiding questions to ensure consistency across participants. All responses were documented in writing, and recurring themes were later analyzed using inductive thematic analysis by two independent researchers.

The study was conducted in accordance with the Declaration of Helsinki and was approved under decision no. 112/04.03.2022 by the Ethical Board of the Faculty of Physical Education and Mountain Sports, Transilvania University of Brașov.

### 2.4. Therapist Qualifications and Intervention Principles

The therapeutic swimming program was implemented by a multidisciplinary team composed of APA instructors/swimming therapists, behavioral therapists, and a supervising clinical psychologist. All aquatic sessions were delivered in a 1:1 format, ensuring each child received individualized attention and support throughout the program. The swimming therapists were graduates of Kinetotherapy and Special Motricity, a university-level program focused on motor rehabilitation, therapeutic movement, and physical intervention strategies for individuals with disabilities. In addition to their academic training, all instructors had completed specialized certification in therapeutic swimming, with an emphasis on aquatic interventions for children with neurodevelopmental disorders. Each had accumulated more than three years of hands-on experience working directly with children with special needs in clinical, educational, or NGO settings. Their work was supported and continuously observed by behavioral therapists during each session to ensure both physical and emotional safety.

The behavioral component of the intervention was reinforced by behavioral therapists with academic degrees in Psychology, who had completed formal specialization training in (ABA). Each ABA therapist had more than three years of field experience, working within NGOs dedicated to the care and support of children with autism and related developmental disorders. These professionals were responsible for shaping behavioral goals, preparing children during the ABA preparatory phase, and guiding the therapeutic approach used during aquatic sessions.

All components of the swimming intervention were supervised by a licensed clinical psychologist, who reviewed session plans, observed implementation, and held weekly consultations with swimming and behavioral therapists to ensure that intervention principles were followed consistently and ethically.

Intervention Principles

The therapeutic swimming sessions were built upon a combination of ABA strategies, Self-Determination Theory (SDT), and principles from structured teaching. This integrative approach aimed to improve not only motor functioning but also behavioral engagement, self-regulation, attention, and adaptive responses in a highly sensory aquatic environment.

From ABA, the team applied techniques such as prompting, shaping, positive reinforcement, and task analysis. Tasks were broken down into manageable steps, and each step was reinforced immediately upon successful performance. For example, a complex task like jumping into the pool or floating independently was divided into micro-goals, each rewarded with verbal praise, visual approval, or access to a preferred toy. Visual cues and verbal instructions were standardized across therapists to ensure consistency and generalization of learning.

SDT principles were integrated to enhance internal motivation and active participation. Children were given limited but meaningful choices during activities (e.g., choosing between floating tools or selecting specific commands during water play), encouraging autonomy and reducing resistance. Instructors also focused on creating a safe, encouraging, and emotionally responsive environment to strengthen the child’s sense of competence and connection.

Structured teaching strategies were used to introduce predictable routines and visual structure to the sessions. The routine used during the swimming sessions helped children anticipate what was coming next, reducing anxiety and increasing task engagement. The water itself was used strategically to support body awareness, muscle relaxation, joint pressure regulation, and sensory integration. This combination of physical, behavioral, and motivational strategies provided a flexible but consistent framework to meet the diverse needs of children with ASD during the aquatic program.

### 2.5. Intervention: Therapeutic Swimming Program

The swimming intervention was designed to address psychological, cognitive, physical, and social domains. Sessions were conducted individually (1:1 child–therapist) for safety and personalization, with occasional peer activities for socialization. Of the 39 children who began the swimming program, 36 completed the full intervention, resulting in a 92% participation rate. Most participants attended 14 to 16 sessions, with consistently high engagement throughout.

Session Structure and Reinforcement System

Each therapeutic swimming session lasted approximately 45 min and followed a consistent, structured sequence designed to support learning, engagement, and emotional regulation. The sessions were divided into five distinct phases:

1. Warm-up and Transition (5 min): The session began with a calm transition from the locker room to the pool environment. Visual schedules and personalized verbal prompts were used to prepare the child for the session. This phase aimed to establish predictability and reduce anticipatory anxiety. Children were greeted with enthusiasm by their swimming therapist, reinforcing the relational aspect of the intervention.
2. Adaptation Phase (10 min): During this segment, children were supported in entering the water and acclimating to its sensory properties (temperature, buoyancy, resistance). Activities focused on body orientation, supported floating, and exploratory movement. The goal was to help children regulate their responses to tactile and vestibular stimulation while building trust in the instructor and the environment.
3. Core Therapeutic Activities (20–25 min): This was the most intensive part of the session, focused on motor planning, coordination, and adaptive behavior. Activities included obstacle courses, dynamic balance tasks, water games requiring turn-taking, and tasks targeting specific motor goals (e.g., kicking, reaching, floating, diving with support). Skills were selected and adapted based on each child’s functional level. ABA-based strategies like task chaining, modeling, and graduated prompting were used to scaffold participation and build independence. Activities often had a rhythm-based or musical component to increase engagement and attention.
4. Social and Sensory Play (5 min): Children were encouraged to explore the water freely, play with floating toys, or engage in brief social routines (e.g., water dances, clapping games, or sharing floating boards). This phase promoted generalization of learned behaviors in a more relaxed and semi-structured setting. It also supported positive peer modeling if other children were nearby in the pool.
5. Cool-down and Closure (5 min): The session ended with predictable closing rituals, such as calm floating with therapist support, singing a familiar song, or giving “high fives” to mark success. This helped reinforce the child’s sense of achievement and prepare for a smooth transition out of the pool. A short verbal recap was provided to the child and caregiver, highlighting accomplishments and participation.

Reinforcement System

A personalized reinforcement system was implemented for each child based on individual preferences, goals, and communication abilities. The approach was primarily individual, as sessions were conducted 1:1, but followed ABA-based reward principles:

- Immediate reinforcement was provided through verbal praise (“Great job kicking!”), high fives, or access to preferred toys (e.g., balls, floating rings).
- Visual reinforcers were also used (e.g., smiley faces, waterproof visual token boards) to acknowledge task completion.
- In cases where children had a higher level of cognitive and behavioral control, a simple token economy was used. Tokens were earned after each completed task and exchanged at the end of the session for a reward (e.g., extra playtime or a chosen water game).
- Social reinforcers were emphasized during group moments, like brief games involving therapists and peers, although these were secondary to the individual objectives.

The reinforcement plan was adapted weekly based on the child’s progress and emotional state, under supervision of the clinical psychologist, following a predictable format designed to reduce anxiety and support attention [34,35]. Tasks were introduced gradually using chaining and reinforcement strategies [36,37], while allowing children to make simple choices to enhance autonomy and engagement [38].

Each swimming session took place in a therapeutic pool with a depth between 1.1 and 1.4 m, allowing both safety and full range of motion for children aged 8–16 years. Water temperature was maintained between 31–32 °C to provide thermal comfort and promote muscle relaxation. The program included two sessions per week, over eight consecutive weeks, totaling 16 sessions. Each exercise type (adaptation, coordination, attention, and self-confidence) was repeated between 4 and 6 times per session depending on the child’s progress and tolerance. Progression followed a step-by-step model, starting from assisted floating and simple movements during the first two weeks, advancing to independent swimming tasks and cooperative games in the final weeks. Therapists increased task complexity by gradually reducing physical assistance and adding verbal prompts or time-based challenges.

The therapeutic swimming intervention was designed as a structured program that combined physical, behavioral, and sensory goals specific to children with ASD. The general objectives were to enhance motor coordination, promote social interaction, and reduce anxiety through gradual exposure to water-based tasks. Each activity category was associated with distinct mechanisms of action and expected outcomes.

1. Water adaptation and sensory regulation. Sessions began with exercises focused on water familiarization, such as splashing, bubble-blowing, and submersion with therapist support. These activities helped children adjust to tactile and proprioceptive sensations, promoting body awareness and reducing fear or sensory overload. Gradual immersion and guided breathing also encouraged self-regulation and calmness.
2. Motor coordination and balance. Core aquatic exercises—floating, gliding, and supported kicking—were used to strengthen large muscle groups, improve balance, and enhance bilateral coordination. The resistance and buoyancy of water allowed children to perform smooth, repetitive movements, supporting proprioceptive feedback and joint stability.
3. Attention and focus. Structured task sequences, such as collecting floating objects or following visual markers, required sustained concentration and response to verbal cues. These activities were informed by behavioral principles derived from ABA, reinforcing correct performance through praise and short breaks.
4. Learning and cognitive engagement. Progressive swimming tasks, including stroke imitation and timed challenges, targeted sequencing, memory, and rule-following. These cognitive demands were expected to strengthen executive functions and increase compliance with therapist instructions.
5. Self-confidence and social interaction. Cooperative games and paired tasks (e.g., passing a ball, synchronized kicking) were introduced during the second half of the program. These activities created opportunities for shared attention, turn-taking, and emotional expression. Success in mastering swimming skills was used as positive reinforcement to increase self-efficacy and motivation.

Together, these exercise categories provided both physical conditioning and structured behavioral learning opportunities. The water environment’s sensory modulation and therapist-guided feedback were expected to reduce hyperarousal, increase engagement, and support global developmental progress in children with ASD.

Intervention objectives:

- Psychological/Emotional: enhance self-confidence, reduce anxiety, increase social trust.
- Cognitive/Behavioral: improve attention, support self-regulation, reduce hyperkinetic behaviors.
- Physical/Motor: improve balance, coordination, strength, and endurance; ensure water safety.
- Social/Functional: encourage group participation, improve communication, transfer gains to daily life.

Intervention means and methods

Intervention Practices

- Progressive exposure to water: shallow activities, floating, structured swimming.
- Play-based therapy with games, songs, and aquatic objects for motivation.
- Routine and repetition to provide predictability.
- Positive reinforcement through verbal praise and rewards.

Intervention materials and equipment

- Floatation aids (kickboards, noodles, arm floats).
- Balls of various sizes and colors.
- Rings and diving toys for underwater retrieval.

**Figure 2:**
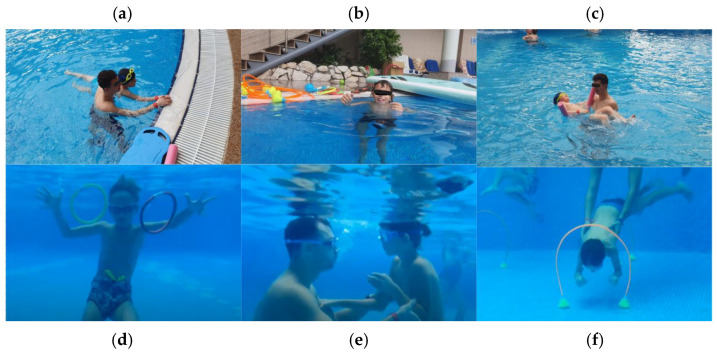
Intervention specific exercises. (**a**) Surface adaptation and entry—Therapist supporting child at the pool edge for safe entry and water confidence. (**b**) Object retrieval and water orientation—Child reaching for floating toys near pool edge to practice eye-hand coordination and orientation. (**c**) Floating and control—Child holding a floating toy, guided by therapist to practice front and back float. (**d**) Underwater motor control—Child diving underwater with rings, practicing immersion and breath control. (**e**) Underwater eye contact, breathing and social engagement—Therapist and child facing each other underwater, practicing breathing, attention, imitation, and nonverbal interaction. (**f**) Swimming through hoops—Child swimming underwater through hoops, practicing motor planning, breath regulation, and directional swimming. Source: Author’s own work.

Intervention specific exercises (Figure 2):

- Adaptation & Warm-up: shallow walking, bubble blowing, supported floating, wall-kicking.
- Motor coordination & Strength: kickboard flutter kicks, arm imitation games, poolside jumps, ball passing.
- Attention & Focus: “follow the leader” swimming, color-coded target retrieval, timed kicking tasks.
- Self-confidence & Social Play: relay games, cooperative ball play, independent short swims, underwater gates.
- Relaxation & Cool-down: floating with support, gentle kicking with noodle, stretching, breathing exercises.

Anticipated challenges of the intervention included variability in participants’ autism severity and associated conditions, which could influence responsiveness to therapeutic swimming program; fluctuations in attendance due to family or health factors; communication barriers that could limit children’s ability to follow instructions; and safety concerns related to impulsive behaviors or hyperkinetic tendencies in the pool environment. These challenges were addressed using visual supports, gradual water exposure, structured routines, and continuous therapist supervision to ensure both safety and therapeutic engagement.

### 2.6. Statistical Analysis

Data were analyzed using IBM SPSS Statistics version 26. Descriptive statistics (mean, standard deviation, and confidence intervals) were calculated for each of the five questionnaire subscales at the three time points (T − 8, T0, and T + 8). Internal consistency of the Parent Perception Questionnaire (PPQ-Autism-Swim) was examined using Cronbach’s alpha, and test–retest reliability was assessed with intraclass correlation coefficients (ICC).

Repeated-measures ANOVA was performed to evaluate changes over time within subjects across the three assessments. When significant main effects were found, Bonferroni-adjusted post hoc comparisons identified pairwise differences. Effect sizes were expressed as partial eta squared (η^2^), which estimates the proportion of total variance in the dependent variable associated with the factor, within the sample. It should be interpreted as an indicator of effect magnitude, not as a measure of causal variance explained. All analyses were conducted per protocol, including only participants who completed the full 8-week intervention and provided complete questionnaire data. Statistical significance was set at *p* < 0.05 for all analyses. For descriptive purposes, mean change scores between the three assessment points (T − 8, T0, and T + 8 weeks) were also calculated for each subscale. These values represent simple within-subject mean differences and are presented to illustrate the direction and magnitude of change before the inferential tests. However, statistical significance of these changes was determined exclusively through the repeated-measures ANOVA reported, included only for descriptive clarity. Prior to conducting repeated-measures ANOVAs, assumption checks were performed. Normality of the distribution was evaluated using the Shapiro–Wilk test, and Mauchly’s test of sphericity was applied to assess the assumption of sphericity. In cases where sphericity was violated, the Greenhouse–Geisser correction was applied. Effect sizes for paired comparisons were calculated using Cohen’s d.

## 3. Results

At baseline, the study included 39 children with ASD, aged between 8 and 15 years. Of the 39 children enrolled, three discontinued during the 8-week intervention period (two due to scheduling conflicts and one due to medical issues unrelated to the intervention), resulting in 36 children completing the full therapeutic swimming program.

Parent-reported questionnaires were collected from 64 parents across the three assessment points (T − 8 weeks, T0, and T + 8 weeks). These responses represented both parents for most children, with some families providing only one parent response. Mothers accounted for most participants, while fathers comprised the remainder. All parents confirmed that they spent more than eight hours per day with their child, ensuring adequate familiarity with the child’s daily functioning and behavior to provide reliable responses. The final analysis therefore included questionnaire data from 36 children, supported by reports from 64 parents.

Reliability analysis from Table 1 confirmed good to excellent internal consistency for the Parent Perception Questionnaire (PPQ-Autism-Swim). At baseline (T − 8 weeks), Cronbach’s α values were 0.82 for General Well-being, 0.84 for Physical Activity & Energy, 0.78 for Attention & Focus, 0.80 for Learning & Cognitive Progress, and 0.86 for Self-Confidence & Social Behaviors. The overall scale showed α = 0.91, indicating high internal reliability. Test–retest reliability, calculated in a subsample of ten parents, produced intraclass correlation coefficients (ICC) ranging from 0.82 to 0.89 across the five subscales, confirming stable responses over time. Subsequent assessments (T0 and T + 8) maintained high reliability, with overall α values increasing from 0.92 to 0.94.

Table 2 presents descriptive mean changes for each subscale across the three assessments, summarizing within-subject score differences. These values were included for descriptive clarity only, while statistical significance was determined through the repeated-measures ANOVA. At baseline (T − 8 weeks), parent-reported scores across all domains were low to moderate, with averages ranging from 2.33 in Attention & Focus to 2.63 in General well-being. The relatively high coefficients of variation (11–15%) reflected substantial differences between children at this stage.

Between T − 8 and T0, parents reported higher scores across all subscales. These changes may reflect ongoing ABA routines, maturation, or shifts in parent perception. Average scores increased by about half a point, particularly in general well-being, learning progress, and attention and focus. These score differences likely reflect developmental changes occurring during this period, rather than the influence of any specific therapeutic component. From T0 to T + 8, parents continued to report higher scores across all domains, though these reports cannot be attributed to swimming alone. Means increased by approximately one point compared to T0, with scores rising to between 3.85 and 4.15 across subscales. These changes were highly significant (*p* < 0.001) and were most notable in physical activity & energy and self-confidence & social behaviors. Parents consistently reported reductions in hyperkinetic behaviors, greater emotional regulation, and more frequent engagement in social interactions.

Overall, by the end of the intervention period, 35 out of 36 children showed measurable progress, and coefficients of variation decreased below 10%, indicating not only improvement at the individual level but also greater consistency in outcomes across the group. Effect sizes (Cohen’s d) were calculated for significant time effects and are presented further, together with the results of the repeated-measures ANOVA.

Table 3 shows the results of the repeated-measures ANOVA for each of the five questionnaire subscales. Across all domains, there was a highly significant main effect of time (all F values > 120, *p* < 0.001), indicating that children’s scores improved significantly between the three assessment points. The size of these effects was very large, with partial η^2^ values ranging from 0.78 to 0.82, indicating that after accounting for other sources of variance, between 78% and 82% of the remaining variance in the dependent measures was associated with the time factor. Across all five subscales, the repeated-measures ANOVA indicated very large effects of time, with partial η^2^ values ranging from 0.78 to 0.82. These results suggest that the intervention explained between 78% and 82% of the variance in outcomes. Parents reported the largest perceived changes in Physical Activity & Energy (partial η^2^ = 0.82) and Self-Confidence & Social Behaviors (partial η^2^ = 0.81), while Learning & Cognitive Progress (partial η^2^ = 0.78) and Attention & Focus (partial η^2^ = 0.79) also demonstrated strong effects. Taken together, these effect sizes confirm that the therapeutic swimming intervention had a substantial and consistent impact across all domains.

Post hoc pairwise comparisons confirmed a stepwise progression:

- from T − 8 weeks to T0, after ABA therapy, children showed significant improvements across all subscales (*p* < 0.001).
- from T0 to T + 8 weeks, following the swimming intervention, further significant improvements were observed (*p* < 0.001).
- the overall comparison from T − 8 weeks to T + 8 weeks was also highly significant for all subscales (*p* < 0.001), confirming strong cumulative effects.

These descriptive patterns show that parent-reported scores increased over time, but the design does not allow the specific influence of ABA or swimming to be isolated. The results describe parent-reported changes over time, which may reflect the natural progression of development, ongoing therapeutic routines, or the way parents perceived their child’s experience during the swimming sessions. Because no control group was included, causal mechanisms cannot be inferred, and the study does not determine whether therapeutic swimming itself produced the changes reported by parents. Statistical tests identify score variation across time points, but these should not be interpreted as evidence of treatment effects. Statistical tests identify score variation across time points, but these should not be interpreted as evidence of treatment effects.

### 3.1. Qualitative Feedback

The thematic analysis of parental interviews revealed five interrelated domains of perceived change: general well-being, energy and physical activity, attention and focus, learning and cognitive progress, and self-confidence and social behaviors.

Parents frequently described children as calmer, happier, and more emotionally balanced, often using phrases such as “more relaxed” or “less frustrated.” Several mentioned that children seemed to enjoy water activities so much that they anticipated the next session with excitement, which improved mood stability at home.

In terms of physical activity and energy, parents reported improved endurance, posture, and general motivation to move. Some noted that their children were “more willing to go outside” and “less fatigued after play.”

Regarding attention and focus, parents described better listening and task engagement, even outside the pool environment. They attributed this partly to the structured nature of swimming sessions and the need to follow instructions closely.

For learning and cognitive progress, parents observed enhanced memory and willingness to try new challenges. They also noticed more spontaneous imitation and understanding of rules in daily routines.

Finally, self-confidence and social behaviors were among the most frequently mentioned themes. Parents noted more initiative in group play, increased eye contact, and pride in newly learned swimming skills. Many said the children’s visible success in the pool had transferred to other settings, improving self-esteem and willingness to communicate.

Together, these qualitative findings complement the quantitative results, providing a richer understanding of how parents perceived the therapeutic swimming experience and its broader influence on their children’s behavior.

The themes were interpreted within a developmental-behavioral framework, emphasizing how structured physical activity and sensory-motor experiences can facilitate emotional regulation, social competence, and adaptive learning in children with ASD.

## 4. Discussion

This research should be interpreted as an exploratory descriptive study. The design does not permit conclusions about effectiveness, and no causal associations between the aquatic program and observed changes can be inferred. The findings reflect parent perceptions within a broader therapeutic trajectory that included an ABA phase prior to swimming.

This study examined the impact of an eight-week therapeutic swimming intervention, following a period of ABA therapy, on the psychological, behavioral, and physical outcomes of children with ASD. The results demonstrated significant improvements across all five domains assessed by parent-reported questionnaires: general well-being, physical activity and energy, attention and focus, learning and cognitive progress, and self-confidence and social behaviors. “Parent reports indicated perceived changes in emotional well-being, attention, and social engagement over the study period, although these cannot be attributed specifically to the swimming sessions.” However, given the lack of a control group and reliance on parent-reported outcomes using a non-validated instrument, these findings should be interpreted with caution. The observed changes cannot be attributed with certainty to swimming alone. It is also possible that some effects reflect expectancy bias, as parents who had already invested time and effort in the ABA phase may have held positive expectations for any subsequent therapy. Additionally, halo effects may have influenced parental ratings, especially in a context where therapists maintained close communication with families. These factors underscore the exploratory nature of the study and the need for more rigorous controlled trials using objective outcome measures to evaluate the independent and additive effects of therapeutic swimming in multidisciplinary interventions. At the end of the intervention, 35 of the 36 children showed measurable progress, with reductions in hyperkinetic behaviors [39,40,41], enhanced emotional regulation, greater confidence, and increased social participation [42,43]. Qualitative feedback from parents confirmed these findings, highlighting improvements in calmness, task focus, sleep quality, and pride in newly acquired abilities.

Our findings are consistent with prior research emphasizing the benefits of therapeutic swimming program in children with ASD [44,45]. Similar improvements in emotional regulation, self-confidence, and social engagement following aquatic interventions were also reported by Caputo et al. (2018), Zanobini and Solari (2019), and Alaniz et al. (2017) [10,11,12]. These mechanisms are consistent with findings by Marzouki et al. (2022), Ansari et al. (2021), and Vonder Hulls et al. (2006), who showed that the warm, supportive aquatic environment promotes sensory integration, rhythmic motor control, and intrinsic motivation in children with ASD [14,15,44].

Each outcome dimension can be linked to specific mechanisms supported by prior research. Improvements in general well-being likely resulted from the calming and rhythmic nature of aquatic movement, which promotes sensory regulation and reduces physiological stress [15,44]. Warm water and hydrostatic pressure stimulate parasympathetic activity, helping children reach a more relaxed emotional state.

Gains in physical activity and energy are consistent with evidence that aquatic exercise enhances endurance, balance, and muscle tone while minimizing joint strain [9,15]. The buoyancy and resistance of water encourage continuous movement, improving strength and coordination in children with ASD.

The observed progress in attention and focus may stem from the structured and repetitive nature of swimming tasks, which combine visual, auditory, and proprioceptive cues. This multimodal stimulation supports sensory integration, reducing distractibility and improving sustained attention [10,11].

Learning and cognitive progress may be explained by the integration of motor learning and behavioral reinforcement. Swimming tasks often require sequencing, imitation, and rule-following, which stimulate executive functions and procedural memory [23]. Repetition and positive reinforcement help consolidate these cognitive processes.

Finally, improvements in self-confidence and social behaviors likely result from the mastery of new motor skills in a supportive and enjoyable environment. Success in swimming builds self-efficacy, and cooperative aquatic play increases opportunities for social interaction and communication [10,13].

Previous studies have reported improvements in motor coordination, physical fitness, and self-regulation following aquatic interventions found that swimming enhanced social engagement and reduced stereotypical behaviors in autistic children [46,47,48]. The present study extends this evidence by combining quantitative measures with qualitative parental feedback, illustrating not only improvements in measurable domains but also meaningful changes in family routines and social functioning. Compared with land-based interventions, therapeutic swimming program appears to provide unique sensory and motor feedback that supports both physical and psychological development. Furthermore, the stepwise design of this study, with ABA preceding swimming, highlights how behavioral and physical interventions can be complementary, reinforcing outcomes across domains. We acknowledge the use of a non-standardized parent-report tool and thematic interviews as potential limitations. However, strong reliability metrics and methodological rigor in qualitative analysis strengthen the credibility of the findings. Furthermore, explicitly using ABA outcomes as a reference point helped clarify the added contribution of swimming.

The significance of these results extends beyond the immediate therapeutic benefits and highlights broader implications for clinical practice, educational strategies, and health policy in Romania and internationally. While therapeutic swimming program and therapeutic swimming has been increasingly recognized in Western countries, its integration into rehabilitation systems in Eastern Europe remains fragmented. Our findings suggest that therapeutic swimming is not only feasible in the Romanian context but also highly effective in supporting developmental progress in children with ASD.

The current results align with international evidence showing that aquatic environments provide unique opportunities for children with ASD to engage in structured, enjoyable, and motivating activities. For example, Caputo et al. (2018) and Zanobini & Solari (2019) [10,11] both documented significant improvements in social interaction and communication following therapeutic swimming program programs. Similarly, Marzouki et al. (2022) [15] demonstrated that aquatic training enhanced both physical coordination and psychosocial functioning in children with autism. Our study confirms these findings while contributing novel data from an underrepresented region. By using a mixed-methods approach (quantitative questionnaires and qualitative parental interviews), we capture not only measurable developmental gains but also the lived experiences of families, thereby broadening the ecological validity of our conclusions.

An important feature of this study is its design, which situated the swimming program after a period of ABA therapy. This sequencing provided an opportunity to evaluate how physical activity might consolidate and extend behavioral gains. The results indicate a stepwise progression: children improved following ABA but showed further and more pronounced improvements after engaging in therapeutic swimming. The design does not allow conclusions about interactions or comparative effects between ABA and swimming, reinforcing skills such as self-regulation, attention, and social participation in naturalistic contexts. Such complementarities are consistent with ecological models of child development, where interventions in one domain (behavioral training) can be reinforced by experiences in another (physical activity).

Such complementarities are consistent with Bronfenbrenner’s ecological systems theory (1979) and Newell’s ecological model of motor development (1986) both of which emphasize the dynamic interaction between individual characteristics, the environment, and the task [25,26]. In this framework, behavioral training (ABA) and physical activity (therapeutic swimming) act as interdependent systems that reinforce each other: structured learning supports compliance and self-regulation, while aquatic practice provides a sensory-rich context for applying and generalizing those skills.

Several mechanisms may account for the improvements observed. The aquatic environment reduces gravitational load, provides proprioceptive and tactile stimulation, and creates opportunities for repetitive, rhythmic movement—all of which may regulate arousal and support sensory integration.

Moreover, the structured yet playful nature of swimming sessions may increase motivation and engagement compared to land-based therapies that some children perceive as demanding. The ritualized structure of sessions (warm-up, skill training, cool-down) may also contribute to predictability and routine, which are particularly beneficial for children with ASD who often struggle with transitions. These differences can be understood through the lens of sensory integration theory [27] which posits that controlled sensory stimulation can improve regulation and attention in children with developmental disorders. The aquatic environment provides constant proprioceptive and vestibular input, promoting calmness and sustained engagement. Studies [49,50] by Fragala-Pinkham et al. (2014) and Yilmaz et al. (2004) demonstrated that water buoyancy and hydrostatic pressure reduce gravitational stress and allow smoother movement patterns than those typically achieved in land-based physical activities. This supports the idea that aquatic settings offer a unique context for practicing controlled motion and body awareness.

Beyond child-level outcomes, the study emphasizes the importance of parental perceptions. Families reported that their children not only demonstrated progress in therapy but also carried these gains into everyday life, such as improved sleep quality, better school participation, and enhanced family interactions. These findings underscore that therapeutic swimming does not merely benefit individual children but also alleviates caregiver burden and strengthens family well-being. At a community level, programs such as the one described here could reduce social isolation and provide families with inclusive, supportive environments.

Despite its promise, implementing therapeutic swimming in Romania faces challenges. Access to appropriate pools, trained staff in APA, and integration with healthcare or educational systems remain limited. Costs, geographical disparities, and cultural perceptions of disability may further restrict participation. These barriers highlight the need for policy initiatives that promote inclusive sports and rehabilitation programs at national and regional levels. Investment in training specialists and building infrastructure would be crucial steps toward scaling such interventions.

The PPQ-Autism-Swim played a practical role in capturing parent-observed changes across multiple developmental domains. It was developed in collaboration with local behavioral therapists to reflect everyday functional outcomes relevant to families and practitioners in Romania. While it lacks formal validation, its structure allowed consistent tracking of perceived progress following the swimming program. The tool also helped identify changes that may not be easily captured by clinician-administered instruments, particularly in community-based, real-world contexts. Future studies should build on this by adapting or validating parent-report tools aligned with standardized measures.

### 4.1. Limitations

The study has several important limitations. All quantitative outcomes rely on parent-report, which introduces expectancy effects and subjective interpretation of child behavior. ABA exposure varied widely across participants and was not standardized or monitored, making it a major confounding factor that limits interpretation of score changes. Participants also showed substantial heterogeneity in comorbidities, cognitive levels, communication abilities, and behavioral functioning, which may have influenced both responsiveness to the aquatic program and parent perceptions. The aquatic intervention did not include formal fidelity or adherence checks, restricting our ability to evaluate consistency across instructors and sessions. Additionally, only children who were already able to follow structured tasks were included, introducing selection bias and limiting generalizability to children with higher support needs. Dropout information has now been reported, but missing data and attrition may still influence the interpretability of these descriptive trends. Future studies should aim to use matched control groups, include retention assessments at several follow-up points, and stratify participants by age and experience level to better understand moderating effects.

Another important limitation concerns the assessment instrument. The Parent Perception Questionnaire (PPQ-Autism-Swim) was developed specifically for this study, and although reliability indices were high, formal validation was not performed. The questionnaire’s validity currently relies on expert review and pilot testing rather than statistical confirmation against established clinical scales. Future research should include standardized tools alongside the PPQ to verify its accuracy and strengthen the interpretability of the findings.

Another limitation concerns the lack of inter-rater analysis between parents. Although both parents were encouraged to respond, in most of the families, both caregivers completed the questionnaire, while in the remaining 8 families, only one parent responded. While this approach provided a broader range of perspectives, the inclusion of both single- and dual-parent reports may introduce variability. Future studies should control respondent structure or analyze agreement between raters when multiple responses are available. The sample size, although larger than some prior pilot studies, remains modest and limits generalizability. The reliance on parental reports introduces potential bias, as perceptions may be influenced by expectations or social desirability. The absence of a control group prevents ruling out maturation effects or external influences unrelated to the intervention. Furthermore, while the questionnaire demonstrated good reliability, it was newly developed and requires further validation in larger and more diverse samples. Finally, heterogeneity among participants (comorbidities such as ADHD, epilepsy, or speech delays) may have influenced responsiveness to the intervention.

While the intervention included multiple structured goals, not all outcomes were operationalized through standardized or direct observational measures. For instance, broader objectives such as behavioral flexibility, social participation, and emotional self-regulation were captured only through parent-reported outcomes. These constructs would benefit from future studies using direct behavioral observations, third-party assessments, or validated clinical tools to improve objectivity and comparability.

Finally, the findings should be interpreted in light of the Romanian context, where access to adapted aquatic interventions is limited and services are primarily delivered through NGOs. These contextual factors may affect both implementation and outcomes. Replication in other cultural and institutional settings is necessary to evaluate the generalizability and broader applicability of the program.

### 4.2. Practical Implications

The findings offer several practical recommendations for aquatic therapists working with children with ASD.

1. Use consistent structure and routines. Each session should begin and end with predictable greetings, warm-up, and closure rituals to reduce anxiety and support behavioral regulation.
2. Maintain optimal water conditions. Keep the water temperature between 31–32 °C and use a pool depth that allows therapists to provide support safely (about 1.1–1.4 m).
3. Integrate behavioral reinforcement. Combine ABA principles with aquatic activities by using clear instructions, verbal praise, and immediate positive feedback after successful tasks.
4. Promote gradual progression. Start with simple movements (floating, wall kicks) and increase task complexity only when the child shows readiness. Avoid sudden transitions or overstimulation.
5. Encourage social play. Incorporate cooperative games such as ball passing or relay tasks to build communication, turn-taking, and shared attention.
6. Collaborate with psychologists and parents. Share observations regularly and align swimming goals with each child’s behavioral and therapeutic objectives.

## 5. Conclusions

This exploratory study suggests that parents perceived meaningful changes across the intervention period. These preliminary findings highlight the need for controlled studies to examine whether therapeutic swimming contributes independently to child outcomes. Parents described the swimming sessions as a structured environment in which their children engaged in motor, attentional, and social activities, although these perceptions cannot be linked to specific intervention effects. By adapting the aquatic environment and tailoring instruction to individual needs, swimming provides not only physical exercise but also opportunities for learning, inclusion, and confidence-building. In this sense, therapeutic swimming extends the role of adapted physical education by integrating health promotion, behavioral support, and psychosocial development within a single activity. Such evidence reinforces the importance of incorporating aquatic programs into adapted physical education curricula, ensuring that children with autism have access to meaningful, enjoyable, and developmentally appropriate opportunities for growth.

This study offers preliminary descriptive information about parent-reported changes during an adapted swimming program. in the therapeutic program and development of children with autism spectrum disorder. Over the course of the program, parents reported changes across emotional, attentional, learning-related, social, and behavioral domains over the study period, with 35 out of 36 participants showing measurable improvement. The parent-report questionnaire proved to be a reliable and valuable instrument for capturing these multidimensional changes, offering both quantitative data and insight into everyday functioning as perceived by families.

This study provides preliminary insights into the potential benefits of integrating therapeutic swimming after ABA therapy in children with ASD. While parent-reported outcomes suggested improvements across emotional, behavioral, and physical domains, the findings must be interpreted cautiously due to the study’s exploratory design, small sample size, absence of a control group, and reliance on a non-validated instrument. These results should be seen as a foundation for further research rather than definitive evidence. Future studies should prioritize controlled trials, larger samples, and the formal validation of the PPQ-Autism-Swim tool to establish reliability and comparability with standardized assessments.

Importantly, the adapted swimming intervention addressed several challenges commonly encountered in autism therapy. Difficulties such as hyperkinetic behaviors, reduced self-confidence, and limited social interaction were alleviated through structured aquatic exercises, positive reinforcement, and the unique sensory environment of the pool. The challenges of heterogeneity, communication barriers, and variability in daily functioning were overcome in part by combining behavioral therapy with a consistent, engaging, and inclusive physical activity program.

Because ABA exposure varied across participants and no control group was included, the study cannot isolate or attribute changes to the swimming program. The findings should therefore be interpreted as descriptive observations of parent-perceived changes within a broader therapeutic trajectory Taken together, the findings suggest that therapeutic swimming, supported by validated assessment tools, can play a central role in the holistic care of children with autism. By integrating adapted therapeutic swimming program into multidisciplinary treatment plans, practitioners may help children achieve not only clinical gains but also meaningful improvements in daily life and family well-being.

## Figures and Tables

**Table 1:** Reliability of the Parent-Report Questionnaire across time points.

Subscale	Time Point	α	95% CI		ICC (Baseline Test–Retest, n = 10)
			Lower	Upper	
General Well-being	T − 8 w	0.82	0.76	0.88	0.85
	T0	0.84	0.79	0.89	
	T + 8 w	0.86	0.81	0.91	
Physical Activity & Energy	T − 8 w	0.84	0.79	0.89	0.87
	T0	0.85	0.80	0.90	
	T + 8 w	0.87	0.83	0.91	
Attention & Focus	T − 8 w	0.78	0.71	0.85	0.82
	T0	0.80	0.73	0.87	
	T + 8 w	0.82	0.76	0.88	
Learning & Cognitive Progress	T − 8 w	0.80	0.73	0.87	0.83
	T0	0.82	0.76	0.88	
	T + 8 w	0.84	0.79	0.89	
Self-Confidence & Social Behaviors	T − 8 w	0.86	0.81	0.91	0.89
	T0	0.88	0.84	0.92	
	T + 8 w	0.90	0.87	0.93	
Overall Scale	T − 8 w	0.91	0.88	0.94	0.88
	T0	0.92	0.89	0.95	
	T + 8 w	0.94	0.92	0.96	

Note: α—Cronbach’s α value, CI 95%—Confidence interval 95%; ICC—Intraclass Correlation Coefficient.

**Table 2:** Descriptive statistics by subscale.

Subscale	Time Point	X	SD	Min	Max	95% CI		CV%	*p*	d
						Lower	Upper			
General Well-being	T − 8 w	2.63	0.30	2.0	3.2	2.53	2.73	11.4	-	
	T0	3.13	0.33	2.6	3.8	3.02	3.24	10.5	<0.001	2.87
	T + 8 w	4.05	0.31	3.4	4.5	3.94	4.16	7.7	<0.001	
Physical Activity & Energy	T − 8 w	2.60	0.32	2.1	3.3	2.49	2.71	12.3	-	
	T0	3.20	0.35	2.7	3.9	3.08	3.32	10.9	<0.001	2.75
	T + 8 w	4.15	0.34	3.6	4.6	4.03	4.27	8.2	<0.001	
Attention & Focus	T − 8 w	2.33	0.35	2.0	3.0	2.21	2.45	15.0	-	
	T0	2.93	0.38	2.6	3.7	2.80	3.06	13.0	<0.001	2.33
	T + 8 w	3.85	0.41	3.4	4.5	3.71	3.99	10.6	<0.001	
Learning & Cognitive Progress	T − 8 w	2.50	0.34	2.0	3.2	2.38	2.62	13.6	-	
	T0	3.00	0.36	2.5	3.6	2.88	3.12	12.0	<0.001	2.60
	T + 8 w	3.95	0.37	3.5	4.4	3.83	4.07	9.4	<0.001	
Self-Confidence & Social Behaviors	T − 8 w	2.45	0.37	2.1	3.1	2.32	2.58	15.1	-	
	T0	3.05	0.36	2.6	3.7	2.92	3.18	11.8	<0.001	2.58
	T + 8 w	4.02	0.39	3.6	4.5	3.89	4.15	9.7	<0.001	

**Note:** X—mean, SD—standard deviation, Min—minimum, Max—maximum, CI 95%—confidence interval, CV%—coefficient of variation, *p*—significance threshold, d—Cohen’s d value.

**Table 3:** Repeated-measures ANOVA and post hoc comparisons by subscale.

Subscale	F	*p*	Partial η^2^	Post Hoc (T − 8 vs. T0)	Post Hoc (T0 vs. T + 8)	Post Hoc (T − 8 vs. T + 8)
General Well-being	142.3	<0.001	0.80	<0.001	<0.001	<0.001
Physical Activity & Energy	158.7	<0.001	0.82	<0.001	<0.001	<0.001
Attention & Focus	133.9	<0.001	0.79	<0.001	<0.001	<0.001
Learning & Cognitive Progress	125.4	<0.001	0.78	<0.001	<0.001	<0.001
Self-Confidence & Social Behaviors	151.2	<0.001	0.81	<0.001	<0.001	<0.001

Note: F—Anova value, *p*—significance threshold, η^2^—effect size.

## Data Availability

The data presented in this study are available on request from the corresponding author. The data are not publicly available due to privacy data included.

## Abbreviations

The following abbreviations are used in this manuscript:

ABA	Applied Behavior Analysis
ADHD	Attention-Deficit/Hyperactivity Disorder
ADOS-2	Autism Diagnostic Observation Schedule, Second Edition
ANOVA	Analysis of Variance
ASD	Autism Spectrum Disorder
DSM-5	Diagnostic and Statistical Manual of Mental Disorders, Fifth Edition
ICC	Intraclass Correlation Coefficient
PPQ-Autism-Swim	Parent Perception Questionnaire for Autism Swimming Program

## Appendix A

### Parent-Report Questionnaire on Child Progress

Background Information (to be completed by the parent/guardian)

Child Information
Child’s code/ID (assigned by researcher): ___________
Age: ______ years
Sex: □ Male □ Female □ Other
Diagnosis: _____________________________________
Comorbidities (if any): □ ADHD □ Epilepsy □ Intellectual Disability □ Speech Delay □ Other: ____________
Current therapies: □ ABA □ Speech Therapy □ Occupational Therapy □ Other: ____________

Family Information
Parent/Guardian completing the questionnaire: □ Mother □ Father □ Both □ Other (specify): _______
Parent’s age: ______ years
Parent’s education level: □ Primary □ Secondary □ University □ Postgraduate
Time spent daily with child: □ <4 h □ 4–8 h □ >8 h
Siblings: □ None □ 1 □ 2 □ 3+

Consent
I confirm that I am the parent/guardian of the child listed above and that I have spent sufficient daily time with my child to provide informed and accurate responses in this questionnaire.

Signature: _______________________ Date: _____________

Instructions: Please indicate how often the following statements apply to your child in the past week.

Response scale: 1 = Never | 2 = Rarely | 3 = Sometimes | 4 = Often | 5 = Always

Subscale 1: General well-being
My child appears calm and relaxed during daily activities.
My child shows enjoyment when engaging in familiar routines.
My child seems less anxious or irritable compared to before.
My child adapts well to daily transitions (e.g., home to school).

Subscale 2: Physical activity and energy
My child participates willingly in active play or exercise.
My child has enough energy to complete daily activities.
My child shows improved coordination and movement control.
My child enjoys swimming or other physical activities.

Subscale 3: Attention and focus
My child can stay focused on a task without getting distracted.
My child listens attentively when given instructions.
My child can finish an activity without leaving it incomplete.
My child shows better concentration during therapy or learning.

Subscale 4: Learning and cognitive progress
My child demonstrates progress in learning new skills.
My child remembers and applies what he/she has learned.
My child follows step-by-step instructions more accurately.
My child is able to solve simple problems or tasks independently.

Subscale 5: Self-confidence and social behaviors
My child shows pride when completing a task successfully.
My child initiates social interactions more often.
My child is more confident when trying new activities.
My child communicates more effectively with peers or adults.
